# Ecology of Anti-Biofilm Agents II: Bacteriophage Exploitation and Biocontrol of Biofilm Bacteria

**DOI:** 10.3390/ph8030559

**Published:** 2015-09-09

**Authors:** Stephen T. Abedon

**Affiliations:** Department of Microbiology, The Ohio State University, 1680 University Dr., Mansfield, OH 44906, USA; E-Mail: abedon.1@osu.edu; Tel.: +1-419-755-4343; Fax: +1-419-755-4327

**Keywords:** bacteriophage ecology, biocontrol, biofilms, biofilm control, biofilm eradication, ecology, phage ecology, phage therapy

## Abstract

Bacteriophages are the viruses of bacteria. In the guise of phage therapy they have been used for decades to successfully treat what are probable biofilm-containing chronic bacterial infections. More recently, phage treatment or biocontrol of biofilm bacteria has been brought back to the laboratory for more rigorous assessment as well as towards the use of phages to combat environmental biofilms, ones other than those directly associated with bacterial infections. Considered in a companion article is the inherent ecological utility of bacteriophages *versus* antibiotics as anti-biofilm agents. Discussed here is a model for phage ecological interaction with bacteria as they may occur across biofilm-containing ecosystems. Specifically, to the extent that individual bacterial types are not highly abundant within biofilm-containing environments, then phage exploitation of those bacteria may represent a “Feast-or-famine” existence in which infection of highly localized concentrations of phage-sensitive bacteria alternate with treacherous searches by the resulting phage progeny virions for new concentrations of phage-sensitive bacteria to infect. An updated synopsis of the literature concerning laboratory testing of phage use to combat bacterial biofilms is then provided along with tips on how “Ecologically” such phage-mediated biofilm control can be modified to more reliably achieve anti-biofilm efficacy.

## 1. Introduction

It is frequently argued that bacteriophages (phages), the viruses of bacteria, are the most abundant category of microorganisms on Earth [1], while their hosts are probably at least tied for most abundant cellular organisms [2,3,4,5]. Furthermore, it is often suggested that the predominant state that bacteria exist, in nature, is as biofilms [6,7]. This includes the commensal bacteria that are associated with organisms such as ourselves, with biofilm formation also often accompanying chronic bacterial infections [8,9,10,11]. Though not subject to nearly as much study as the interaction between phages and planktonic or broth-grown bacteria, the sheer numbers of phages and biofilm-associated bacteria present in environments is suggestive that phage-biofilm interactions are ecologically important, such as, for example, in terms of phage-mediated horizontal gene transfer between bacteria, *i.e.*, transduction [12].

A biofilm forms in the course of microorganism adherence to other microorganisms, either to sister conspecifics or instead to other bacteria that are already biofilm associated. The resulting biofilms typically though not always are associated with surfaces. Even more prominently [13], bacteria found in biofilms display some form of matrix consisting of one or more type of extracellular polymeric substance (EPS). Matz [14], in distinguishing biofilms from planktonic bacteria, goes further, summarizing biofilms in terms of (p. 199) “*Adherence to a substratum; self-encasement into an extracellular matrix; life at high cell densities; and differentiation resulting in population heterogeneity*.” 

Presented here is an updating of a model of how phage exploitation of biofilm bacteria in nature might occur [15,16,17,18], especially in terms of the population dynamics of phages, their infection of bacteria, and associated lytic cycles. Considered in a companion article is the inherent ecological potential of bacteriophages *versus* especially antibiotics to serve as anti-biofilm agents [19]. Despite a resulting potential for phages to remove bacterial biofilms at least locally over microscales, it is clear given the ubiquity of biofilms found within various environments that phage action on its own is not sufficient to result in complete biofilm eradication from environments. This issue is relevant to the use of phages as deliberately applied anti-biofilm agents if in fact biofilm eradication is a goal. That is, phage presence in and of itself generally is insufficient to completely remove harmful or nuisance biofilms from surfaces even given the use of phages to which target bacteria are sensitive, e.g., [15].

A growing literature considers phages as therapeutic or biocontrol [20,21] agents of pathogenic or other bacteria, e.g., [22,23], and the use of phages specifically as anti-biofilm agents has been recently reviewed by a number of authors and publications [15,18,24,25,26,27,28,29]. In addition to discussing the ecology of phage-biofilm interactions, considered as well are related issues of phage-mediated biocontrol or phage therapy of biofilm-associated bacteria. Provided specifically is a synopsis of the phage-treatment-of-biofilms literature and then tips on how to optimize, in terms of phage application alone, the use of viruses of bacteria as anti-biofilm agents. Overall, arguments are provided that (1) phages inherently may be more effective anti-biofilm agents than antibiotics [19], (2) phages though presumably capable of exploiting biofilm-associated bacteria in the wild nevertheless are not well equipped to completely eliminate their bacterial-biofilm prey on their own, and (3) a growing body of literature provides evidence that inherent phage anti-biofilm activity nonetheless often can be harnessed to effectively combat chronic bacterial infections. Phages in nature, that is, probably should not be viewed as biofilm eradicators or as biofilm-formation-preventing agents but instead, for those phages so adapted, as opportunistic biofilm exploiters. Consistently achieving phage-mediated biofilm eradication in particular likely requires overall greater phage impact on biofilm bacteria than phages in nature may be able to achieve.

## 2. Theory of Phage-Biofilm Ecological Interaction

Provided in a companion article [19] is a logical argument along with extensive supporting material for why phages, or at least a subset of phages, may serve ecologically as more effective antagonists to otherwise intact biofilms than many naturally occurring small-molecule antibacterials such as antibiotics. To summarize the argument: (1) Lytic phages are predators in the sense that they kill to obtain resources from victim bacteria, thereby supplying strong evolutionary motivation for phages to develop highly effective bacteria-killing abilities. (2) Phages are able to spatially concentrate their antibacterial activity against target bacteria in a manner that may not be as readily accomplished using naturally occurring, small-molecule antibacterials, especially ones that impact target bacteria from an extracellular source. Particularly, while antibiotic pharmacokinetics tends to be mostly independent of densities of sensitive bacteria, phage concentrations tend to be enhanced the more target bacteria that are present [16,30,31]. (3) Phages directly disrupt biofilm structure, and at a minimum this is accomplished through the lyses of the bacteria that they infect. Though not addressed here, note that temperate phages in the course of lysogenic cycles also can substantially impact and be impacted by biofilms ecologically, an issue discussed at some length in Abedon [15].

Despite phage advantages as anti-biofilm agents, understanding how to fully exploit phages towards biofilm eradication can require an appreciation of the context in nature of phage bactericidal actions, where phages are under natural selection to survive and compete with other phages rather than specifically to disrupt bacterial biofilms. To achieve such disruption, such as within clinical settings, it therefore can be necessary to modify phage-biofilm interactions from what instead naturally can or would occur. Considered in Section 3 are some means by which phage-mediated anti-biofilm activity may be so modified. Provided first, however, is a scenario for the natural ecology of phage-biofilm interactions.

### 2.1. Underlying Assumptions

Bacteriophage interactions with biofilm bacteria in many ways are equivalent to their interactions with planktonic bacteria, such as can be approximated in the laboratory within well-mixed broth. Differences arise, however, that are associated especially with (1) spatial heterogeneity that can be present due to the association of biofilms with discontinuously located surfaces, (2) spatial structure that results from impediments to environmental mixing, (3) likely clonal clumping of bacteria within biofilms such as forming microcolonies, (4) greater heterogeneity over short spatial scales of bacterial physiologies as are typically observed in biofilms *versus* bacteria that are not associated with biofilms, and also, perhaps, (5) reduced rates or potentials for phage virions to directly penetrate to all bacteria associated with a given biofilm. Considered in this section in greater detail are a number of assumptions that are pertinent to the development of a conceptual model of phage-biofilm ecological interactions, particularly in terms of phage population dynamics across both micro and macro scales within biofilm-containing environments. Of these assumptions, two or three are relevant to both planktonic and biofilm bacteria (particularly the first and last, *i.e.*, see Section 2.1.1 and Section 2.1.4, but also to a degree the second, Section 2.1.2) and one is pertinent especially to phage-biofilm interactions (the third, Section 2.1.3). 

#### 2.1.1. There are Phages to Which Biofilm Bacteria are Sensitive

Biofilm bacteria can be resistant to phages but so too can planktonic bacteria [32,33]. For phage therapy to be effective it is always necessary to identify phages to which target bacteria are sensitive. Therefore, we can take as a premise that, in terms of the population dynamics of phage interactions with biofilm-associated bacteria, there will tend to exist one or more phage types that in fact will be capable of productively infecting a given biofilm-associated target bacterium. Extensive studies of phage-biofilm interactions in the laboratory are suggestive that such phages exist for either some or many biofilm-forming bacteria [15,18,24,25,26,27,28,29]. See also Table 2, below, which summarizes the phage biocontrol of biofilms literature, and see too, for example, recent speculation by Friman and Buckling [34] on the phage potential to replicate in association with biofilms. By contrast, any bacteria, either planktonic or biofilm associated, which are not susceptible to any of the phages that are located within their immediate environment will not be subject to phage-mediated predation. The resulting lack of phage predation pressure could possibly allow for selection of biofilm formation in bacteria [16].

#### 2.1.2. Bacteria Availability can be Insubstantial across Macroscopic Environments

Bacteria of specific phage susceptibility types are those that are found within a given phage’s host range [32]. As found within biofilms—other than biofilms as associated for example with chronic infections of animals [8]—bacteria of specific susceptibility types will be expected to be relatively rare. This in part, e.g., [35,36], could be due to phage-associated predation pressure, *i.e.*, “Kill the winner” [1,37,38], which can prevent relatively small numbers of bacterial phage susceptibility types from dominating environments. Rarity of specific bacterial types also can be due to the presence of a high diversity of microenvironments within which dissimilar bacterial ecotypes may differentially thrive.

Biofilms themselves can be relatively rare and therefore well separated, such as found coating surfaces that also may be rare within especially pelagic aquatic environments. Furthermore, movement within environments may be slowed by poor mixing, *i.e.*, as can be seen with soils and sediments. Thus, within many environments biofilms can be either rare or difficult to reach as well as individually diverse in terms of what types of bacteria are present. As a result, specific biofilm targets for phage exploitation can—especially in a temporal sense—be few and far between. This assumption, as noted, likely is less applicable to biofilm bacteria associated with chronic infections such as of animals [8].

#### 2.1.3. Bacteria Availability can be Substantial across Microscopic Environments

Biofilm-associated bacteria of specific phage susceptibility types can be present at high densities over shorter spatial scales even as they are found at low densities averaged over larger spatial scales. Specifically, bacteria can be clonally clumped such as into cellular arrangements or microcolonies. This clumping is most obvious within single-species biofilms as the entire biofilm can be viewed as consisting of a single, concentrated mass of clonally related bacteria. Numerous biofilm studies, however, indicate the existence of morphologically distinct regions within, for example, mixed species biofilms that often are referred to as microcolonies, e.g., [7,8,13,39,40,41,42]. Furthermore, the observation of bacterial cellular arrangements is extremely common and these too represent regions of higher bacterial densities as measured over smaller spatial scales, though bacteria found within cellular arrangements are not necessarily associated with bacterial biofilms.

Clumps of bacteria can give rise to disseminating bacteria that are at least temporarily less clumped, and those disseminating bacteria will exist at lower local bacterial population densities than the bacterial clumps from which they arise. That is, holding total bacteria numbers constant, cells that are spatially isolated from one another will exist at lower local cell densities than will clumps of bacteria that have not been spatially isolated from one another; here “local” refers, for example, to spatial scales that are moderately larger than the volume of a single cell,. These different states represent, respectively, *random* dispersions of organisms (spatially isolated) *versus clumped* dispersions (multiple regions where cells are not spatially isolated). In terms of clumped dispersions, bacterial *clumps* consisting especially of clonally related bacteria can serve as regions of high bacteria availability to bacteriophages [16].

#### 2.1.4. Higher Bacteria Availability Should Support more Robust Phage Population Growth

All else held constant, we have an expectation that two or more bacteria of a specific type that are found at higher local densities will be able to support faster as well as more substantial local phage population growth than two or more bacteria of a specific type that are found at lower local densities [16]. Phages as exploiters of specific bacterial types therefore may be supported at lower phage densities by lower bacterial densities, as averaged across environments, while simultaneously these same phages may be locally supported at higher phage densities in the course of their sequential infection of clumps of clonally related target bacteria. Phages furthermore can be viewed as opportunists and/or as displaying feast-or-famine population dynamics, with opportunities, or feasts, associated with phage acquisition of bacteria while “famine”—here as equivalent to low metabolic activity—is the case prior to such acquisition. With biofilms as targets, phage opportunities/feasts may be associated especially with clonal clumps of phage-sensitive bacteria.

### 2.2. General Ecological Scenario for Phage-Biofilm Interactions in Nature

With the above assumptions in mind, we can consider a general model for the population dynamics of phages whose hosts predominantly consist of biofilm-forming bacteria [15,17]. In fact, we can consider three scenarios: (1) Phage-bacterial steady states, *i.e.*, where average phage and bacterial population densities across a given environment are not changing over time; (2) phage populations that are declining in density over time across a given environment; and (3) phage populations that are increasing in density over time. The two latter scenarios are simple conceptual extensions of the first scenario, that of a phage-bacterial steady state. Therefore, we will both begin with and concentrate on the steady state case. It is important to keep in mind in considering the resulting model that steady states are not “Magic”, nor even ideals, but instead are assumptions, *i.e.*, a way of thinking about a system, in this case one in which various parameters happen to balance over macroscopic scales.

#### 2.2.1. Phage-Bacterial Steady States

At steady state across a single environment both phage and bacterial densities are not changing. This does not mean that environments are homogeneous nor that changes in phage or bacterial densities cannot occur locally. Instead, any increases in one location are balanced by decreases in another. If this balance is not present then the phage population density is either increasing or decreasing across the environment, and so too the density of bacterial hosts may be increasing or decreasing. In assuming a steady state within a given environment, then the gain of any one phage will on average be associated with the loss of another phage. Phage gains will occur in association with successful infection of bacteria, and phage losses will occur as phages fail to successfully acquire new bacteria. On average, at steady state, all of the phages released in a given phage-infection burst (gains) will therefore become inactivated (losses), except for one. Each surviving progeny phage on average will therefore replace each surviving parental phage and the phage “Effective” burst size—which is the number of phage progeny produced by a single infection that succeed in establishing a new infection [43]—will be equal to 1 (epidemiologically, this is equivalent to a basic reproductive number, *R*_0_, also of 1). For a phage population that is increasing in size, the effective burst size instead will be greater than 1. For a phage population that is decreasing in size the effective burst size will be less than 1.

These scenarios are valid whether or not bacteria display clumped dispersions and whether or not environments display impediments to mixing, that is, whether or not they display spatial structure. At a steady state within well-mixed environments, such as can be seen given more oligotrophic conditions even in simple one-phage, one-bacterium systems, both bacteria and phage densities can remain more or less constant over time [44]. This constancy can occur particularly given bottom-up control, that is, where predator-prey population dynamics are impacted more by intrinsically low prey availability than by predator influence on prey prevalence [45]. With bacterial clumping, however, localized regions of phage population increase will be broader than simply individual phage-infected bacteria, instead encompassing local regions in which more-rapid phage adsorption and infection of bacteria can occur. Such regions consisting of concentrated phage population growth are referred to here as infection foci. These infection foci are partly analogous to phage population growth at it occurs in the laboratory within phage plaques [15,30]. Infection foci, rather than increasing in number, may display steady states across environments that are a consequence especially of intrinsically low prey availability. Alternatively, increases in numbers of infection foci could result from higher availability of bacterial prey while decreases in numbers of infection foci instead may be associated with lower bacterial availability. 

#### 2.2.2. Infection Foci Steady States

For randomly dispersed bacteria within non-spatially structured environments we still have regions of enhanced phage production. These, though, will consist of isolated, individual, phage-infected bacteria. There also will likely be regions of enhanced phage inactivation, including as found between target bacteria—and given heterogeneity among target bacteria, either physiologically or in terms of phage-resistance mechanisms, phage inactivation can occur as well upon bacterial infection. At steady state, phage gains within the regions of production are exactly balanced by phage losses within regions of inactivation: A phage is “Born” and then, more often than not, given a steady state, a phage will “Die” prior to successfully infecting a host bacterium. When bacteria display instead clumped dispersions, then the local dynamics of phage gains—that is, within a plaque-like focus of infection—can involve not just individual, isolated bacterial infections. These dynamics instead also may involve rapid movement of newly formed phage virions across, e.g., micrometer scales to not-yet infected, locally available, phage-sensitive bacteria.

The ecological scenario within which a biofilm-exploiting phage exists thus is one in which natural selection favors not just the productivity of individual phage infections but also, potentially, the productivity of a focus of infection. That is, of primary importance may be the collective output of localized regions of multiple phage infections, ones which are analogous in their spatial structure to phage plaques as found in the laboratory [46]. The phages so produced, notwithstanding their similarity to phage plaques, ideally should be capable of diffusing not only within but also away from the focus of infection within which they were produced. Thus, populations of biofilm-exploiting phages collectively consist of those that are currently infecting individual bacteria, those that are diffusing locally within biofilms as at least approximately equivalent to within phage plaques, and those that have escaped from the focus of infection that produced them. The latter display movement that, ideally for the phages involved, is eventually towards new locations, ones where new infection foci may be founded.

At steady state for infection foci the number of such foci across an environment by definition is assumed to remain constant. Thus, no matter how many phages are produced locally within these environments, that is, particularly as within infection foci, at steady state on average only one of the phages generated by a focus of infection will survive to initiate a new focus of infection (or more if infection foci are increasing in number or less if infection foci are decreasing in number). This scenario, as noted, requires that phages which are capable of infecting biofilm-associated bacteria exist (Section 2.1.1); that these bacteria are relatively rare or at least not highly available within environments (thereby resulting in bottom-up control of phage-bacteria predator-prey dynamics rather than top-down; Section 2.1.2); that the target bacteria for these phages display a clumped dispersion, e.g., as cellular arrangements or bacterial microcolonies (Section 2.1.3); and that phage population growth can locally occur at a faster rate over microscales as a consequence of this clumping (Section 2.1.4). Furthermore, the steady state scenario is equivalent to the epidemiology of an infectious disease where *R*_0_ equals 1, that is, with a phage focus of infection equivalent to a parasite- or pathogen-infected individual. An infection focus, that is, may be viewed from an epidemiological perspective as equivalent to an animal or a plant which is found within a population of susceptible individuals. Indeed, a phage focus of infection, as equivalent to a phage plaque, itself can be viewed as a localized infectious “Disease” [47] of biofilm where chronic shedding of the infectious agent, from the focus of infection, follows a short period of incubation that is equal, minimally, to the latent period of the infection-focus initiating phage infection.

### 2.3. Parallels between Infection Focus and Biofilm Formation 

**Figure 1:**
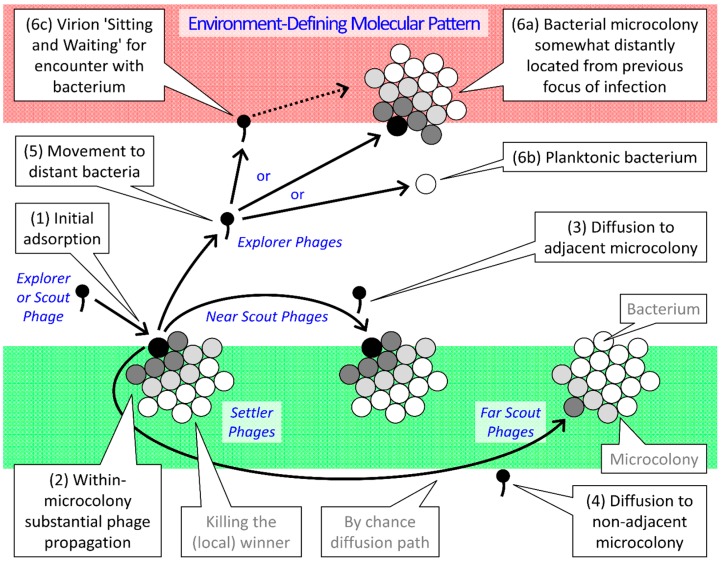
Ecological scenario of phage interaction with biofilm-associated bacteria, figure as derived in part from that of Abedon [15,17]. Most of the bacteria depicted are shown associated with clonal microcolonies. These in turn are embedded within an “Environment-defining molecular pattern” (EDMP), such as animal-produced mucus, but perhaps also including EPS (EDMP or EPS is shown as shaded boxes, green/bottom for “Start”, red/top for “Finish”). A substantial distance, on the order of millimeters, centimeters, meters, or longer, is assumed to exist between the two shown shaded regions. The scenario begins (1) with the “Initial adsorption” of a bacterial microcolony by a phage, either “Explorer” or “Scout” phage as considered further below. “Settler” phages are those that by chance infect bacteria that are associated with their parental microcolony, *i.e.*, as within the same EPS matrix as the parental infection, and these infections can result (2) in substantial phage reproduction. “Scout” phages are those that by chance infect bacteria associated with neighboring rather than the same microcolonies, that is, as found outside of EPS matrix associated with the parental microcolony. Movement of these phages outside of EDMP or EPS, as depicted, nevertheless does not necessarily occur to a substantial extent, but arrows nonetheless are presented thusly for clarity. This movement (3) can be to adjacent microcolonies (either a smaller gap or instead no gap between microcolony EPS matrices) or (4) to more-distantly located microcolonies, both as found within the same biofilm (larger or multiple gaps). “Explorer” phages escape from their parental biofilm (equivalent to traversing a very large gap between EPS matrices) and, if they are lucky, (5) succeed in (6a) encountering microcolonies or instead (6b) encountering individual, especially planktonic bacteria that are found some distance from the parental focus of infection. Particularly the former (6a) may be found in non-parental biofilms, or alternatively macroscale distances away from the parental locus within the same biofilm, e.g., multiple millimeters or more. No phenotypic or genotypic differences, besides the path of their diffusion and location of subsequent bacterial adsorption, otherwise exist between these different phage “Types”. Within a single biofilm these processes can result in phage plaque-like population growth, or formation of what can be described as a focus of infection. Within microcolonies a progression of phage population growth also can occur, starting with an initial adsorption and then followed by further “Active” phage penetration into the same microcolony. Shown as well (6c) is a potential for some phages to bind to EDMP, such as mucus as described by Barr *et al.* [49,50]; see also [51]. This binding may allow phages to wait for bacteria to come to them rather than phages diffusing to bacteria, described here as phages “Sitting and Waiting”. These phage virion interactions with molecular patterns may be reversible, allowing for more localized phage searching for bacteria to infect such as within bacterial biofilms (dotted arrow), though such interactions also might locally slow virions during their searches within biofilms for bacteria to infect.

The development of a single-species biofilm at a minimum involves, e.g., [48], the transition of a founding bacterium from a planktonic state to the initial stages of actual biofilm development (“Attachment”), the post-attachment development of the biofilm (“Maturation”), and subsequent conversion from the biofilm state to some form of bacterial dissemination including as planktonic bacteria (“Dispersion”). The process of phage exploitation of biofilm bacteria, particularly given acquisition of multiple bacteria in the guise of bacterial microcolonies or arrangements found in single micro-location [16], arguably follows an equivalent progression; see Figure 1 for a conceptual model of this scenario of phage-biofilm interactions.

To start, note in Figure 1 the initial or primary phage adsorption event. The initial infection of biofilm-associated bacteria follows the diffusion of a phage virion especially from outside of the biofilm. The resulting virion attachment thus is conceptually equivalent to the surface “Attachment” step of biofilm development itself, which also begins with movement, in this case of a cell, towards the to-be-colonized surface. Indeed, this phage, designated in the figure as “Explorer or Scout Phage” (middle, left), explicitly is attaching to a surface, one consisting of a bacterium, and is doing so in a specific, adaptive manner, just as we can assume is the case given surface attachment by cells to initiate biofilm formation.

The next step of phage interaction with biofilm bacteria, as analogous to biofilm formation itself, involves the development of a focus of infection. This process comprises what can be described as “Secondary” infections as defined in an epidemiological sense [52]: bacterial infections that are spatially as well as temporally distinct from the initial, that is, primary “Attachment” adsorption. The occurrence of secondary infections is conceptually equivalent to the “Maturation” step of biofilm formation in that population growth is occurring, as so too is a potential for heterogeneity: Cells may become infected that display differing physiologies [53] or phages otherwise may display variation in their physiologies such as in terms of the phage processes of lysis inhibition, lysogeny, or pseudolysogeny [15,17,54,55].

Figure 1 differentiates among virions in terms of their contribution to the “Maturation” of a focus of infection. These virions include those that go on to infect bacteria that are immediately adjacent to their parental infection, *i.e.*, bacteria making up the same microcolony or cellular arrangement or which otherwise are encased within what effectively is the same EPS as the parental infection (here termed “Settler” phages). By contrast are those virions that instead diffuse within the same biofilm to close-by phage-sensitive clumps of bacteria, particularly as found within microcolonies or cellular arrangements that are found adjacent to the parent microcolony (“Near Scout” phages). In addition are those phages that diffuse even further within the same biofilm to also encounter phage-sensitive but more distant bacterial clumps (“Far Scout” phages). It is important to keep in mind with these designations, however, that no genotypic or even phenotypic distinction is being made between these different phage “Types” other than in terms of the distances that they diffuse prior to encountering new, phage-sensitive bacteria. These distinctions instead are made in order to differentiate ecological roles, including in terms of the different infection variants that may result. For example, settler phages should be more likely to initiate lysogenic cycles, due to multiple adsorptions of individual bacteria [56], than will far scout phages. Furthermore, the penetration of phages into bacterial microcolonies [57], in the course of what can be described as an active penetration [30], should be a function particularly of the action of settler phages in terms of endogenously generated phage virions. See Table 1 for overview.

**Table 1:** Proposed phage ecological types associated with biofilm exploitation.

Type	Gap Length ^1^	Ecological as well as Infection-Focus Developmental Roles
Settler	No gap	Attachment; Virions exploit the same microcolony as that of their parental infection
Near Scout	Smaller or none	Maturation; Virions initiatiate the exploitation of individual bacterial microcolonies ^2^
Far Scout	Larger or multiple	Maturation; Virions form the leading edge of individual infection foci
Explorer	Very large	Dispersion; Virons diffuse out of infection focus to found new infection foci

^1^ Distance between EPS matrices associated with individual bacterial microcolonies; ^2^ It is conceivable that scout phages may also reach microcolonies that other virions have already reached.

One additional phage “Type” is indicated in Figure 1 and Table 1 and that is the “Explorer” phage. Explorer phages have the distinction of moving out of the biofilm proper to potentially initiate new infection foci some distance from the parental focus. It is this process of explorer phage release from a biofilm that is equivalent to the biofilm stage of “Dispersion”. For phages, however, the equivalent “Dispersion” step does not likely follow the “Maturation” step in a well-defined manner. Instead, from the first phages that are released from infections of biofilm bacteria to the last, all have some potential to diffuse out of the biofilm and therefore serve as explorers. This likelihood, however, may increase as the phage-sensitive bacteria that are associated with a focus of infection decline in number, such as due to phage-induced lysis. Indeed, this phage-induced lysis can be viewed as a further aspect of the “Maturation” of a phage focus of infection within a biofilm. Thus, while biofilm or microcolony maturation should coincide more or less with an increase in surface-associated biomass, a phage focus of infection, due to phage-induced lysis as well as additional hydrolytic activity, should by contrast coincide with a decline in surface-associated bacterial biomass. Indeed, more generally, a key phage ecological function is their contribution to decomposition within environments [58], particularly to initiation of the mineralization of what prior to phage infection instead are intact bacteria.

In natural environments, explorer phages may go on to found new infection foci, or instead may become inactivated following their diffusion out of biofilms. Similarly, disseminating bacteria, following dispersion, may become inactivated. Indeed, given a rarity of colonizable surfaces, or of potential host bacteria, then the likelihood of either phage or bacterium surviving to complete their attachment-maturation-dispersion-attachment life cycles likely is low. For phages, given that the dissemination step is difficult, and assuming that the number of explorer phages that are generated at any given time within an environment as a whole is not large, the result can be a low potential for phages to completely eradicate biofilm-forming bacteria from environments, at least as a consequence of the action of natural processes alone. Similarly, the potential for a phage focus of infection to eliminate all of the immediately available bacteria found in their immediate midst also may not reach 100%. As a consequence of these issues, there is a reasonable potential for phages that specialize on biofilm-forming bacteria, along with phage-sensitive biofilm-forming bacteria that they specialize on, to coexist. This is rather than phages driving their bacterial hosts to extinction, at least locally, or otherwise going extinct themselves.

### 2.4. Phage-Biofilm Coexistence

We can expect from the above model—just as we do within well-mixed environments—that phage infections of bacteria will be relatively rare so long as phages and/or target bacteria also are relatively rare. Furthermore, and potentially more specific to biofilms, heterogeneity in the ability of target bacteria to support individual phage infections, particularly due to physiological differences, could result in less efficient phage population growth as well as less effective localized eradication of target bacteria than may be the case given more physiologically homogeneous bacterial targets. With regard to bacterial eradication within biofilms, however, Harper *et al.* [26] suggest that phage infections of even physiologically quiescent bacteria ultimately may become lethal to the bacteria; similarly, see [54,59,60,61]. Heilmann *et al.* [62] by contrast argue in favor of the existence of bacterial states within biofilms that can serve as refuges from phage attack (p. 12829): “What is required for long-lived coexistence [of phages and bacteria] on the edge of bacterial refuges is merely that the bacteria in the center of the colony are so resilient that phage cannot sustain themselves in there, whereas recently divided bacteria on the edge of the colonies are (possibly very) susceptible to phage infection.” Across a given biofilm or instead across the diversity of possible biofilms, it is possible that all of these various scenarios to some degree are correct. Stable coexistence of phages and bacterial biofilms within environments thus can likely occur as a consequence of biofilm and/or specific bacteria rarity (*i.e.*, bottom-up control) as well as the existence of bacterial refuges within biofilms and, re: Harper *et al.* [26], and others [54,59,60,61], bacterial refuges from phage attack within biofilms are not necessarily absolute.

In more complex ecosystems, phage-bacterial dynamics may not be what determines the potential for phages to coexist with bacteria. Instead, and for various reasons as summarized above, populations of biofilm-forming bacteria may not display sufficient densities to support an overwhelming phage presence. That is, the total phage numbers or peak phage densities generated across environments is not going to be large if bacteria, phage infections, or foci of phage infections are rare. As a result, the potential for phages to eliminate populations of target bacteria across environments, or even across individual biofilms, likely will tend to be low. This is just as herbivores existing at lower population densities, such as may occur due to predation (*i.e.*, top-down control), tend to be poorly equipped to denude environments of plant species [63]. The inherent potential of specific phages to exploit biofilm-associated bacteria as predators [19] nevertheless may provide an advantage to phages over antibiotics as anti-biofilm agents. In practical terms, however, and just as with antibiotics, we therefore cannot assume that simply the presence of these bacterial antagonists will result in the elimination of biofilms. Such eradication may be enhanced, however, particularly to the extent that the concentration of phages—locally, more globally, and/or over time—can be increased to beyond those levels that may be expected to occur based solely on typical endogenous processes.

Notwithstanding these caveats, under certain circumstances complete biofilm eradication might be possible even without the help of additional factors, such as the anti-microbial action of an animal’s immune system or co-application with antibiotics. This potential nevertheless contrasts with the results of especially *in vitro* studies in which phages have been used against biofilms but without complete biofilm elimination (Table 2, below). More consistent achievement of biofilm elimination likely requires proper phage choice, potentially the use of more than one phage type (given bacteria potential to display especially somewhat phage-specific resistance mechanisms), the addressing of possible difficulties associated with phages reaching all targeted bacteria, and also dealing with limitations on the ability of phages to replicate that may be associated, within biofilms, with phage-refractory bacterial physiological states. Particular the two latter issues have not been well studied using *in vitro* systems, though both have been addressed clinically simply by applying phages especially in multiple doses over the course of treatments. In response to apparent discrepancies between clinical and pre-clinical phage therapy approaches, as well as outcomes, in the following section some of the challenges of phage use as anti-biofilm agents are considered and how these challenges to a degree may be overcome.

## 3. Phage-Mediated Biocontrol of Bacterial Biofilms

Chronic infections typically involve biofilms [8,9,10,11] and a substantial literature exists on the use of phages to treat, clinically, chronic bacterial infections. Phage treatment in general has been reviewed extensively, e.g., [64,65,66], and see in particular the numerous clinical reports that have issued from the Hirszfeld Institute of Immunology and Experimental Therapy in Wrocław, Poland, as described and cited in those reviews, e.g., [67,68]. Though not all use of phages as anti-biofilm agents occurs within the context of treatment of chronic bacterial infections, including industrial concerns or issues of biofouling, nevertheless the clinical application of phages to treat chronic infections is the most visible of such uses. Based on the phage-use-as-anti-biofilm-agents literature [15,18,24,25,26,27,28,29], we can consider how an understanding of the phage ecology of phage-biofilm interactions potentially may be used to bolster phage anti-biofilm efficacy. For comprehensive discussion of earlier, pre-2011 literature, see especially Abedon [15] as well as the other reviews listed above. See Table 2 for a more current listing of the primary phage-use-as-anti-biofilm-agents literature. In that table studies are differentiated in terms of the target organisms studied as well as the timing of phage addition relative to biofilm development. Particularly, treatment prior to the initiation of biofilm formation is described as “Before” whereas treatment following the initiation of biofilm formation is described as “During”.

**Table 2:** Studies of phage-mediated prevention or eradication of biofilms. Shown only are studies in which biofilm presence and treatment has been explicitly indicated. Distinguished are treatments of already formed biofilms (“During”) from prevention of biofilm formation (“Before”); see [19] for additional discussion of these concepts. To receive a designation of “During”, clear indication of bacterial incubation in association with a surface for at least a number of hours prior to phage application must be explicitly indicated. In a few cases it was not possible to determine whether treatment *versus* prevention had occurred and these are indicated with “Before?” In cases where prevention of biofilm formation and treatment of biofilm formation were both equivalently attempted, just “During” is indicated. Note for the 2014 Belgini *et al.* study that more bacteria were tested than are indicated in the table.

Target Species	Context	Timing	Reference	
*Acinetobacter baumannii*	Microtiter plate	During	Thawal *et al.* (2012)	[69]
*Acinetobacter baumannii*	Microtiter plate	During	Mendes *et al.* (2014)	[70]
*Acinetobacter johnsonii*	Ultrafiltration membrane model	Before	Goldman *et al.* (2009)	[71]
*Aggregatibacter actinomycetemcomitans*	Polystyrene microtiter plate	During	Castillo-Ruiz *et al.* (2011)	[72]
*Arthrobacter soli*	Microtiter plate	Before?	Belgini *et al.* (2014)	[73]
*Bacillus subtilis*	Ultrafiltration membrane model	Before	Goldman *et al.* (2009)	[71]
*Brevundimonas* sp.	Microtiter plate	Before?	Belgini *et al.* (2014)	[73]
*Campylobacter jejuni*	Glass	During	Siringan *et al.* (2011)	[74]
*Citrobacter freundii*	“*Environmental surface, stainless steel, high-density polyethylene plastic, and rubber*”	During	Gong and Jiang (2015)	[75]
*Delftia tsuruhatensis*	Glass; Membrane bioreactor	During	Bhattacharjee *et al.* (2015)	[76]
*Enterobacter agglomerans*	Modified Robbins’ device	During	Hughes *et al.* (1998)	[77]
*Enterobacter cloace*	Glass	During	Tait *et al.* (2002)	[78]
*Enterococcus faecalis*	*Ex vivo* tooth root canal	Before?	Khalifa *et al.* (2015)	[79]
*Enterococcus faecalis*	Microtiter plate	During	Khalifa *et al.* (2015)	[79]
*Escherichia coli*	Polyvinylchloride coupons	During	Doolittle *et al.* (1995)	[80]
*Escherichia coli*	Flow cells	During	Doolittle *et al.* (1996)	[81]
*Escherichia coli*	Modified Robbins’ device	During	Corbin *et al.* (2001)	[82]
*Escherichia coli*	Stainless steel	During	Sharma *et al.* (2005)	[83]
*Escherichia coli*	3-channel flow chamber	During	Moons *et al.* (2006)	[84]
*Escherichia coli*	Pegs in microtiter plates	During	Lu and Collins (2007)	[85]
*Escherichia coli*	Pegs in microtiter plates	During	Lu and Collins (2009)	[86]
*Escherichia coli*	Hydrogel-coated catheters	During	Carson *et al.* (2010)	[87]
*Escherichia coli*	Silicone rubber disks	During	Kay *et al.* (2011)	[88]
*Escherichia coli*	Microtiter plate	During	Chibeu *et al.* (2012)	[89]
*Escherichia coli*	Calgary biofilm device	During	Ryan *et al.* (2012)	[90]
*Escherichia coli*	Microtiter plate	During	Hosseinidoust *et al.* (2013)	[91]
*Escherichia coli*	3-channel flow chamber	During	Moons *et al.* (2013)	[55]
*Escherichia coli*	Silicone Rubber Disks	During	Coulter *et al.* (2014)	[92]
*Escherichia coli*	Microtiter plate	Before	Pei and Lamas-Samanamud (2014)	[93]
*Escherichia coli*	Tissue culture plate	During	Schmerer *et al.* (2014)	[94]
*Hafnia alvei*	“*Environmental surface, stainless steel, high-density polyethylene plastic, and rubber*”	During	Gong and Jiang (2015)	[75]
*Klebsiella pneumoniae*	Microtiter plate	During	Bedi *et al.* (2009)	[95]
*Klebsiella pneumoniae*	Microtiter plate	During	Verma *et al.* (2009)	[96]
*Klebsiella pneumoniae*	Microtiter plate; Glass	During	Verma *et al.* (2010)	[97]
*Klebsiella pneumoniae*	Microtiter plate; Glass	During	Chhibber *et al.* (2013)	[98]
*Klebsiella pneumoniae*	Polycarbonate discs	During	Chhibber *et al.* (2015)	[99]
*Klebsiella pneumoniae*	Microtiter plate	During	Jamal *et al.* (2015)	[100]
*Listeria monocytogenes*	Stainless steel	During	Roy *et al.* (1993)	[101]
*Listeria monocytogenes*	Stainless steel	During	Hibma *et al.* (1997)	[102]
*Listeria monocytogenes*	Stainless steel	During	Soni and Nannapaneni (2010)	[103]
*Listeria monocytogenes*	Stainless steel	During	Montañez-Izquierdo *et al.* (2012)	[104]
*Listeria monocytogenes*	Stainless steel	During	Ganegama Arachchi *et al.* (2013)	[105]
*Listeria monocytogenes*	Stainless steel	During	Chaitiemwong *et al.* (2014)	[106]
*Proteus mirabilis*	Hydrogel-coated catheters	During	Carson *et al.* (2010)	[87]
*Proteus mirabilis*	Microtiter plate; Hydrogel-coated catheters	Before	Lehman and Donlan (2015)	[107]
*Pseudomonas aeruginosa*	Flow cells	During	Doolittle *et al.* (1996)	[81]
*Pseudomonas aeruginosa*	Poly(methyl)methacrylate discs	During	Hanlon *et al.* (2001)	[108]
*Pseudomonas aeruginosa*	Microtiter plate	During	Knezevic and Petrovic (2008)	[109]
*Pseudomonas aeruginosa*	Ultrafiltration membrane model	Before	Goldman *et al.* (2009)	[71]
*Pseudomonas aeruginosa*	Hydrogel-coated catheters	Before	Fu *et al.* (2010)	[110]
*Pseudomonas aeruginosa*	Microtiter plate	During	Ahiwale *et al.* (2011)	[111]
*Pseudomonas aeruginosa*	Silicone rubber disks	During	Kay *et al.* (2011)	[88]
*Pseudomonas aeruginosa*	Microtiter plate	Before	Knezevic *et al.* (2011)	[112]
*Pseudomonas aeruginosa*	Microtiter plate	During	Pires *et al.* (2011)	[113]
*Pseudomonas aeruginosa*	Epithelial-cell monolayer	During	Alemayehu *et al.* (2012)	[114]
*Pseudomonas aeruginosa*	Silicone catheter segment	Before	Liao *et al.* (2012)	[115]
*Pseudomonas aeruginosa*	Microtiter plate	During	Hosseinidoust *et al.* (2013)	[91]
*Pseudomonas aeruginosa*	Microtiter plate; *Ex vivo* tooth root canal	During	Phee *et al.* (2013)	[116]
*Pseudomonas aeruginosa*	Rat implant model	During	Yilmaz *et al.* (2013)	[117]
*Pseudomonas aeruginosa*	Microtiter plate; Glass	During	Zhang and Hu (2013)	[118]
*Pseudomonas aeruginosa*	Water biofiltration systems (anthracite or granular activated carbon)	During	Zhang *et al.* (2013)	[119]
*Pseudomonas aeruginosa*	Silicone Rubber Disks	During	Coulter *et al.* (2014)	[92]
*Pseudomonas aeruginosa*	Microtiter plate	During	Mendes *et al.* (2014)	[70]
*Pseudomonas aeruginosa*	Microtiter plate	Before	Pei and Lamas-Samanamud (2014)	[93]
*Pseudomonas aeruginosa*	Polycarbonate discs	During	Chhibber *et al.* (2015)	[99]
*Pseudomonas aeruginosa*	Pegs in microtiter plates	During	Danis-Wlodarczyk *ett al.* (2015)	[120]
*Pseudomonas aeruginosa*	Microtiter plate; Hydrogel-coated catheters	Before	Lehman and Donlan (2015)	[107]
*Pseudomonas fluorescens*	Inox plate placed in microtiter tray	During	Sillankorva *et al.* (2004)	[121]
*Pseudomonas fluorescens*	Glass	During	Sillankorva *et al.* (2008a)	[122]
*Pseudomonas fluorescens*	Stainless steel	During	Sillankorva *et al.* (2008b)	[123]
*Pseudomonas fluorescens*	Stainless steel	During	Sillankorva *et al.* (2010)	[124]
*Pseudomonas putida*	Polystyrene peg in 96-well microtiter plate	During	Cornelissen *et al.* (2011)	[125]
*Pseudomonas* sp.	Microtiter plate	Before?	Belgini *et al.* (2014)	[73]
*Salmonella typhimurium*	Microtiter plate	During	Hosseinidoust *et al.* (2013)	[91]
*Serratia marcescens*	Modified Robbins’ device	During	Hughes *et al.* (1998)	[77]
*Serratia marcescens*	Polystyrene flasks	Before	Zhang *et al.* (2014)	[36]
*Sphaerotilus natans*	Stainless steel coupons and wire screen	During	Gino *et al.* (2010)	[126]
*Staphylococcus aureus*	Polystyrene microtiter plate	During	Del Pozo *et al.* (2007)	[127]
*Staphylococcus aureus*	Microtiter plate	During	Son *et al.* (2010)	[128]
*Staphylococcus aureus*	Microtiter plate	During	Rahman *et al.* (2011)	[129]
*Staphylococcus aureus*	Microtiter plate	During	Kelly *et al.* (2012)	[130]
*Staphylococcus aureus*	Rabbit wound model	During	Seth *et al.* (2013)	[131]
*Staphylococcus aureus*	Rat implant model	During	Yilmaz *et al.* (2013)	[117]
*Staphylococcus aureus*	Silicone discs	During	Lungren *et al.* (2013)	[132]
*Staphylococcus aureus*	Microtiter plate	During	Alves *et al.* (2014)	[133]
*Staphylococcus aureus*	Sheep model of sinusitis	During	Drilling *et al.* (2014a)	[134]
*Staphylococcus aureus*	Plastic pegs	During	Drilling *et al.* (2014b)	[135]
*Staphylococcus aureus*	Cuffed central venous catheters	During	Lungren *et al.* (2014)	[136]
*Staphylococcus aureus*	Microtiter plate	During	Mendes *et al.* (2014)	[70]
*Staphylococcus aureus*	Microtiter plate	During	Gutierrez *et al.* (2015)	[137]
*Staphylococcus epidermidis*	Catheter	During	Wood *et al.* (2001)	[138]
*Staphylococcus epidermidis*	Hydrogel-coated catheters	Before	Curtin and Donlan (2006)	[139]
*Staphylococcus epidermidis*	Microtiter plate	During	Cerca *et al.* (2007)	[140]
*Staphylococcus epidermidis*	Microtiter plate	During	Gutierrez *et al.* (2015)	[137]
*Staphylococcus lentus*	Stainless steel	During	Sillankorva *et al.* (2010)	[124]
*Vibrio anguillarum*	polypropylene plastic tubes	During	Tan *et al.* (2015)	[141]

### 3.1. Combatting Phage-Bacterial Coexistence

The lower the densities of phages within environments then, all else held constant, the lower their potential to eradicate target bacteria. Given chronically low densities of sensitive bacteria across environments, then densities of phages to which those bacteria are sensitive are expected to be low as well. At the same time, however, it is possible for phage densities to be relatively high locally in association with clumps of such bacteria [16]. To eradicate biofilms it is probable that phage densities need to remain relatively high over all of the biofilm that is being targeted, and for these higher phage densities to persist more or less over the duration of treatment.

It is possible (1) that such high phage densities, both spatially and temporally, can occur as a consequence of natural processes, or (2) that phage treatments need only somewhat reduce biofilm presence to be counted as anti-biofilm treatment successes. In this section what is considered instead are those circumstances in which one or both of those possibilities does not hold along with what to do in terms of phage-mediated biocontrol or phage therapy should that be the case. For more general considerations of issues that might be addressed while “Debugging” phage therapy and biocontrol protocols, particularly in terms of phage therapy pharmacology, see [30,31,142,143,144]. Unless phages are capable of successfully treating biofilms on their own accord, then strategies to consider towards bolstering the biofilm-clearing potential of phages can include the following.

#### 3.1.1. Apply Phages for Longer

In terms of chronic infections, which often involve biofilms, treatment using phages typically will require multiple, repeated dosings or even continuous dosing. Time until treatment success using phages also can be on the order of weeks. It is mainly during proof-of-principle experimentation that single dosing tends to be used in phage therapy protocols. Single-dose treatment with phages, however, is much less the case given actual clinical phage therapy, e.g., such as in terms of treatment of pulmonary infections [66]. As a default assumption, therefore, it can be wise to avoid minimizing dosing repetition even though occasionally single-dose clinical or veterinary treatments have displayed efficacy [145,146].

#### 3.1.2. Apply Reasonable Quantities of Phages

Individual phage doses when treating biofilms need not be exceptionally high, e.g., exposure of biofilms to phage titers of 10^7^/mL appears to be a fairly normal clinical approach to treatment. Natural phage-biofilm infection dynamics are presumably better approximated at such relatively low *versus* higher phage titers, that is, with an expectation that higher titers within the vicinity of target bacteria will be subsequently generated in the course of productive phage infection of target bacteria [19]. In addition, given application of phage cocktails rather than individual phage types [147,148,149,150] there may be less interference between different phages when dosing with such relatively low phage numbers [143]. Dosing at those levels, particularly in combination with repeated or continuous dosing, also might help to foster what can be described as an active penetration of phages into biofilms [30].

Though a somewhat speculative concept, the basic idea of active penetration is that each phage application results in at least primary phage infections along with phage-induced bacterial lysis. Secondary phage infections, as defined in an epidemiological sense, that is, infections initiated by phages produced by primary infections [52], may occur deeper within a biofilm’s structure than primary infections. Alternatively or additionally, phage-induced lysis of outer layers of bacteria making up biofilms might aid the penetration of subsequently applied phages further into biofilms, *i.e.*, as in the course of repeated or continuous dosing.

Higher phage doses, to the extent that they can reduce the productivity of phage infections or the amount of phage-mediated bacterial lysis, might interfere with that progression [30,151]. On the other hand, dosing with too low phage titers might result in insufficient phage progress into biofilms, unless the phages employed are particularly effective at boosting their numbers in the course of interacting with biofilm bacteria. In particular, if one cannot count on the dissemination of phages on their own accord throughout treated biofilms then equivalent phages will need to be exogenously supplied to achieve phage densities *in situ* that are sufficient to eradicate biofilms. In terms of experimentation it can be helpful to test a range of phage titers, including somewhat high titers, e.g., 10^8^/mL final concentrations [55,143] or greater, as well as multiple dosing during the treatment of biofilms, and particularly to do so in the course of initial *in vitro* experimentation *versus* more difficult or expensive *in vivo* or *in situ* testing.

As an aside, note that phage dosing is described here in terms of phage titers rather than phage multiplicities, just as dosing with antibiotics is done in terms of antibiotic amounts rather than in terms of ratios of antibiotic amounts to target bacterium counts. Contrasting what is often the practice in phage therapy publications (e.g., Table 2), dosing is described here in this manner as it often is both confusing and potentially misleading to describe phage dosing using the concept of multiplicity of infection (MOI) rather than in terms of phage titers. In addition, under less controlled circumstances, such as phage therapy as practiced clinically, it often is difficult to determine what the density of target bacteria might be, and typically such determinations of target bacterial densities are not even attempted. See Abedon [15,31,142] for additional discussion of this issue of the use phage titers *versus* phage MOIs in describing phage dosing during phage therapy experimentation.

#### 3.1.3. Biofilm Disruption can Aid Biofilm Clearance

It can be possible to improve phage penetration to biofilm bacteria even beyond that which may be achieved via multiple dosing or active penetration. This can be accomplished via disruption of the biofilm matrix prior to or in conjunction with further antibacterial application. As a result of such disruption, biofilm bacteria may be more readily reached, may be reduced in number by having been removed entirely from the environment, may display improved physiologies for phage infection or antibacterial control, or instead may be transformed to varying degrees to a more planktonic state. Disruption of the biofilm matrix may be accomplished via scraping, debridement of tissues [9], or application of chemicals or enzymes [152,153]. The latter include EPS depolymerases [15,26,154,155], which are typically phage-encoded enzymes that are capable of degrading biofilm matrix.

EPS depolymerases can be phage infection and even virion associated or, at least in principle, can be added in a purified form along with phages. In addition, and particularly towards reducing the expense of enzyme production and purification, the secretion of a variety of biofilm-disrupting enzymes might be engineered into a single, probiotic-type bacterium [153,156]. So-engineered bacteria could then be applied to biofilms to degrade its matrix prior to or otherwise in the course of more traditional antibacterial and/or phage application. The advantage of such an approach, in addition to potentially reducing overall costs, is that EPSs are diverse and engineering multiple EPS depolymerase genes into a single phage could be challenging.

When phages encode their own EPS depolymerases then these enzymes are generated within the biofilm itself during phage infection, which at least potentially could aid in biofilm degradation. In principle, multiple EPS depolymerases also might be supplied individually by different phages making up a single cocktail, and these might even be able to synergistically interact towards bacterial and/or biofilm clearance [150]. Having EPS depolymerases produced solely by phages during infection can result, however, in these enzymes being unavailable immediately following initial dosing using purified phages—or at all if a specific phage producing a particularly EPS depolymerase within a cocktail fails to successfully infect—that is, unless the enzymes are integral components of virions.

#### 3.1.4. If at First you don’t Fully Succeed, try Applying Greater Quantities of Phages

There is a consideration [143] that is applicable particularly to situations where the use of phages alone is contemplated towards reducing numbers of biofilm bacteria, especially to very low levels. This can be either phage therapy of people with compromised immune systems or instead phage-mediated biocontrol within environments in which immune systems are not involved. Here the problem is that once bacterial densities are sufficiently low on surfaces then the ability of those biofilms to support phage propagation will, we can speculate, also be low. At this point, phage treatment may require even greater augmentation to achieve desired levels of biofilm disruption, e.g., repeated dosing with potentially even higher phage titers than may have been effective earlier during treatment regimens. In addition, phage-independent disruption of biofilms at this point may be necessary to the extent that it is those bacteria to which phages have been unable to penetrate that would tend to remain intact even as biofilm presence otherwise declines. In the case of phage therapy of individuals possessing effective immune responses, infections may not need to be reduced microbiologically to zero through phage action alone in order to eliminate the pathogen from the body. As a consequence, this issue of increasing the titer of phage doses over the course of treatments may be less relevant for phage therapy under most circumstances, though perhaps not all.

Phages also can themselves be modified or combined with other substances or procedures in the treatment of biofilms, including in combination with antibiotics. See Harper *et al.* [26] for discussion of additional means by which phage anti-biofilm activity may be enhanced via the use of factors that are in addition to phages themselves.

## 4. General “Pros and Cons” of Phage Therapy

Phage use in terms of their potential utility as anti-biofilm agents is considered above. For the sake of objectivity, in this section various potential shortcomings as well as advantages that have been attributed to the use of phages generally as antibacterial agents are considered. For further discussion, see [157,158,159,160].

Shortcomings, or “Cons” associated with the use of phages as antibacterial agents, particularly in comparison with antibiotics, include the following; (1) Phages possess relatively narrow host ranges which can make presumptive treatment using phages challenging [148,149]. (2) Phages are proteinaceous and so as a consequence, and as with any protein-based “Drug”, have a potential to induce anti-phage adaptive immune responses. (3) Phages via transduction have the potential to move bacterial genes between bacterial hosts, including potential virulence factor genes (though to a degree the typical narrowness of phage host ranges likely limits the contribution of therapeutic phages to horizontal gene transfer between bacteria; see also the typical rejection of temperate phages for therapeutic use). (4) Many otherwise lytic phages are temperate, that is, able to display lysogenic cycles, and these phages generally must be avoided for phage therapy use since, at a minimum, temperate phages upon successful infection are often able to convert phage-sensitive bacteria to phage-insensitive ones. (5) In addition to transducing bacterial virulence factor genes, many phages, particularly temperate ones, can carry these genes as normal components of their genomes, and as a consequence phage full-genome sequencing and annotation are typically required prior to therapeutic use. (6) The size as well as chemical properties of phage virions results in differences in pharmacokinetic behavior relative to typical small-molecule antibacterials. (7) There exist intellectual property issues that are associated with the development of naturally occurring phages as pharmaceuticals. (8) Physician familiarity with phage use is slight in comparison to physician familiarity with antibiotics as antibacterial agents. (9) To date, formal demonstration of either phage safety or therapeutic efficacy has been minimal particularly within the context of European or North American sanctioned clinical trials [64,65,161]. (10) The regulatory framework for phages as drug-like agents is still evolving. (11) Acceptable phages are not always available for all potential target bacteria. For example, non-temperate phages to which the opportunistic pathogen, *Clostridium difficile*, is susceptible have still not be identified despite substantial effort to do so [162], and so too identifying phages which target certain bacterial plant pathogens can be challenging [163].

Alternatively, advantages of phage use as antibacterial agents, that is, “Pros”, can include: (1) Demonstrated safety in use along with a relative absence of pharmacologically emergent properties. That is, phages, once adequately characterized, tend to not possess surprising toxicities or side effects during clinical or experimental therapy. (2) Phages, properly chosen for therapeutic use, are bactericidal rather than bacteriostatic in their antibacterial action. (3) In the course of their antibacterial activity, phages are able to increase their dosage and do so while in direct association with target bacteria. (4) As a consequence of their typically narrow host range, phages tend to have minimal impact on normal flora bacteria. (5) Bacteria which are antibiotic resistant are not also inherently phage resistant. (6) Phages are natural products and are often easily discovered in diverse forms as well as easily characterized in terms of their organismal as well as genomic properties. (7) Phages typically can be readily modified either genotypically or only phenotypically towards alteration of their pharmacokinetic or pharmacodynamic properties. (8) Phages often can be used with other antibacterial agents such as antibiotics as well as disinfectants. (9) The impact of phages on environments, again owing to their narrow host ranges as well as their non-xenobiotic nature, tends to be relatively slight. (10) Phages possess single-hit killing characteristics which in combination with their ability to replicate while in association with target bacteria can allow for relatively low concentration dosing. (11) As noted, phages in many cases appear to be effective anti-biofilm agents.

## 5. Conclusions

Use of bacteriophages to mitigate or eliminate nuisance or pathogenic bacteria affecting bodies or environments can provide multiple advantages in comparison to, for example, the equivalent use of antibiotics [19,157,158]. Whereas antibiotics often can be inefficacious in the treatment of chronic bacterial infections, bacteriophages by contrast have shown a potential for treating even those chronic infections that antibiotic treatments have failed to cure. Phage use against biofilms nonetheless in principle may be improved upon. Refinement of our understanding of phage-biofilm ecological interactions might serve as one path towards identifying approaches that could increase the effectiveness of specific phage anti-biofilm treatments, or lead to more consistent anti-biofilm efficacy.

Successful phage treatment of bacterial biofilms, for example, may require ongoing “Awakening” of metabolically quiescent biofilm bacteria, particularly as infecting phages lytically remove outer biofilm layers. To achieve such layered removal of bacteria, sufficient numbers of phages must be supplied, but—and likely contrasting efforts to eliminate solely planktonic bacteria—not necessarily overwhelming numbers, e.g., such as >>10^8^ phage virions/mL, since very high phage multiplicities of adsorption might interfere with phage infection productivity. At the same time, dosing with too few phages or not dosing often enough can result in reduced efficacy since insufficient phage numbers, unless phages can make up for this deficiency via *in situ* population growth, may serve simply to approximate a natural state of phage-biofilm *coexistence*, where coexistence between phages and target bacteria is not the goal of phage-mediated biocontrol or therapy.

Increased appreciation of phage-bacterial ecological interactions within biofilm-containing environments also could, ideally, inspire greater degrees of experimentation in terms of phage dosing particulars, phage types employed, bacterial strains targeted, or experimental conditions [150,164,165,166,167]. Ideally this might result in more consistent, rapid, or otherwise effective biofilm eradication, as experienced in actual practice, and particularly would be rather than relying on assumptions that phages, as effective predators of bacteria, ought to be able to wipe out sensitive bacteria without substantial regard for dosing or other particulars. At an absolute minimum, it would be extremely useful to the development of phage-based biocontrol generally were studies to seek as end points the eradication of diverse bacterial strains, particularly under realistic treatment conditions, rather than, as often is the case, settling for less useful “Proof-of-principle” results.

In the course of dosing, those phages supplied need to be well matched to their target bacteria in terms of host range [32], as equivalent here to a drug’s spectrum of activity. This spectrum of activity may be manipulated especially by combining phages into cocktails [147,148,149,150]. These phages in most cases will then need to be supplied exogenously, *i.e.*, via dosing, either continuously or instead repeatedly over time. Experience suggests that chronic bacterial infections in fact can require weeks of such treatment to be effective. Nevertheless, bacteriophages are demonstrably able to clear chronic, presumably biofilm-containing infections, though clinically such treatment success in many or most cases likely occurs with immune-system help.

Key to phage-based eradication of bacterial biofilms, given sufficient dosing, is the phage ability to concentrate antibacterial activity explicitly within the bacteria they are infecting. In addition to the resulting bactericidal as well as lytic activity, important as well is the phage ability to “Auto” increase their dosing through population growth within the immediate vicinity of populations or sub-populations of target bacteria, e.g., clonal microcolonies or cellular arrangements. Ultimately, however, an argument may be made that phages can be more effective anti-biofilm agents than antibiotics because with phages the entire biological “Package” of antibacterial activity is what is being delivered to bacterial targets. By contrast, with antibiotic chemotherapy typically only one component of what can be a more extensive antibacterial or anti-biofilm arsenal associated with individual, antibiotic-producing microorganisms [19] tends to be what is applied in the course of anti-biofilm treatment, *i.e.*, just the antibiotic itself.
